# Gone to Pot – A Review of the Association between Cannabis and Psychosis

**DOI:** 10.3389/fpsyt.2014.00054

**Published:** 2014-05-22

**Authors:** Rajiv Radhakrishnan, Samuel T. Wilkinson, Deepak Cyril D’Souza

**Affiliations:** ^1^Department of Psychiatry, Yale University School of Medicine, New Haven, CT, USA; ^2^Abraham Ribicoff Research Facilities, Connecticut Mental Health Center, New Haven, CT, USA; ^3^Schizophrenia and Neuropharmacology Research Group, VA Connecticut Healthcare System, West Haven, CT, USA

**Keywords:** cannabis, psychosis, spice, synthetic cannabinoids, schizophrenia, psychophysiology, schizotypy

## Abstract

Cannabis is the most commonly used illicit drug worldwide, with ~5 million daily users worldwide. Emerging evidence supports a number of associations between cannabis and psychosis/psychotic disorders, including schizophrenia. These associations-based on case-studies, surveys, epidemiological studies, and experimental studies indicate that cannabinoids can produce acute, transient effects; acute, persistent effects; and delayed, persistent effects that recapitulate the psychopathology and psychophysiology seen in schizophrenia. Acute exposure to both cannabis and synthetic cannabinoids (Spice/K2) can produce a full range of transient psychotomimetic symptoms, cognitive deficits, and psychophysiological abnormalities that bear a striking resemblance to symptoms of schizophrenia. In individuals with an established psychotic disorder, cannabinoids can exacerbate symptoms, trigger relapse, and have negative consequences on the course of the illness. Several factors appear to moderate these associations, including family history, genetic factors, history of childhood abuse, and the age at onset of cannabis use. Exposure to cannabinoids in adolescence confers a higher risk for psychosis outcomes in later life and the risk is dose-related. Individuals with polymorphisms of *COMT* and *AKT1* genes may be at increased risk for psychotic disorders in association with cannabinoids, as are individuals with a family history of psychotic disorders or a history of childhood trauma. The relationship between cannabis and schizophrenia fulfills many but not all of the standard criteria for causality, including temporality, biological gradient, biological plausibility, experimental evidence, consistency, and coherence. At the present time, the evidence indicates that cannabis may be a component cause in the emergence of psychosis, and this warrants serious consideration from the point of view of public health policy.

## Introduction

Psychotic disorders are arguably the most serious of mental illnesses, the best known being schizophrenia. As yet, the etiology of schizophrenia and other psychotic disorders remains unclear. There is emerging evidence to support a number of associations between cannabis and psychosis, but the precise nature of these associations remains unclear.

Cannabis is the most commonly used illicit drug by adults, with 18.1 million current users in the U.S. in 2011 (up from 14.5 million in 2007) and ~5 million daily cannabis users (1–3). In the U.S., it was also the most commonly used illicit drug by children 12–17 years (7.9%) in 2011. The age at onset of regular cannabis use appears to be occurring earlier. About 1.3% of eighth graders endorsed daily use of cannabis in 2011 (3). Additionally, the average delta-9-tetrahydrocannabinol (THC) content of cannabis has increased from 3.4% in 1993 to 8.8% in 2008, with concentrations in high potency varieties such as sinsemilla increasing to as high as 11.1% (4). “Medical” marijuana (cannabis) is being legalized increasingly across the U.S. (5, 6). Some states have legalized recreational cannabis use and others are projected to follow suit (7). As a result, individuals, including those with a higher risk for psychosis, who would not have risked the consequences of procuring an illegal drug previously, may now consider exposing themselves to cannabis.

In parallel, there is the emerging phenomenon of the recreational use of Spice, a mixture of synthetic cannabinoids, by young people (8). Among high school seniors, 11.4% reported using Spice in the past year (9). In contrast to THC, the synthetic cannabinoids present in Spice are highly potent full cannabinoid 1 receptor (CB_1_R) agonists (10, 11). There are a number of reports of acute and persistent psychosis immediately following the use of Spice, sometimes with catastrophic outcomes (12–14). In the U.S., emergency department visits related to cannabinoids (149 ED visits per 100,000 population) were second only to cocaine (157.8 ED visits per 100,000 population) (15).

Various lines of evidence point to associations between cannabinoids and psychosis [reviewed in Ref. (16–18)]. These associations may be categorized according to temporal proximity of the onset of psychosis to exposure, duration, and clinical significance of psychosis. Converging lines of evidence suggest that early and heavy exposure to cannabis is associated with a higher risk for psychotic outcomes, including schizophrenia in later life (18–28). In addition, cannabinoids can induce immediate-onset psychotomimetic symptoms that do not persist beyond the period of intoxication (~1 h), as reviewed by us (18). Finally, less well-characterized but perhaps clinically important, cannabinoids are also associated with acute episodes of psychosis that: (1) manifest immediately following exposure, (2) last beyond the period of intoxication, and (3) require clinical intervention (29, 30).

Furthermore, although the associations between cannabinoids and psychosis have gained increasing recognition, the moderators (i.e., variables that affect the direction and/or strength of the relation between an independent, predictor variable – such as cannabis use – and a dependent, outcome variable – such as psychosis) and mediators (i.e., variables that directly account for the relationship between cannabis use and psychosis) are less well-understood. Emerging evidence suggests the crucial role of age of exposure to cannabis (with the period of adolescence being identified as a period of exquisite vulnerability), familial risk, degree of schizotypy, childhood trauma, and the role of genetic factors in moderating this association.

As a preface to this review of the literature, several important issues should be considered. Firstly, cannabis contains more than 70 different cannabinoids (31) of which THC is thought to be the main psychoactive ingredient, while another cannabinoid, cannabidiol (CBD), is thought to have antipsychotic properties (32). THC is hence not the same as cannabis, although most of the experimental studies are conducted using THC. Secondly, cannabis grown in different conditions and different parts of the world has varying potencies based on the content of THC and CBD. The type or potency of cannabis has rarely been accounted for in epidemiological studies. Thirdly, it is important to make a distinction between psychosis as a syndrome and psychosis-like experiences (psychotomimetic effects). While psychosis refers to a heterogeneous group of disorders defined as consisting of positive symptoms (delusions, hallucinations, and thought-alienation phenomena), negative symptoms (alogia, avolition, anhedonia, asociality, and affective flattening), and disorganization/cognitive symptoms (deficits in attention, working memory, problem-solving, and executive function); psychosis-like experiences are characterized by a loss of reality-testing and include derealization, depersonalization, dissociation, hallucination, paranoia, impairment in concentration, and perceptual alterations, which are transient and self-limited. The fact that schizophrenia is a syndrome that is much more than positive symptoms needs to be considered. Negative (e.g., amotivation, asociality, and anhedonia) and cognitive symptoms (e.g., deficits in attention, memory, and executive function) contribute to the disease burden of schizophrenia just as positive symptoms do.

Below herewith, we review existing literature on the association between cannabinoids and psychosis with special focus on the recent critical literature. We categorized major findings into the following categories: immediate psychotic symptoms, psychosis outlasting intoxication, delayed and persistent effects, moderators, and mediators of the association (age of exposure, family history, history of childhood abuse, and genetics), and evidence for causality.

## Immediate and Short-Lived Effects of Cannabinoids

### Non-experimental evidence

#### Evidence from anecdotal reports and surveys of the effects of cannabis

The evidence from anecdotal reports suggests that cannabis may induce acute psychotomimetic effects and precipitate the syndrome of psychosis. One of the earliest systematic studies of the psychotomimetic effects of cannabis was that by the French psychiatrist Jacques-Joseph Moreau (de Tours) in his 1845 book, Hashish and Mental Illness (33). He reported that hashish (cannabis resin) could precipitate “*acute psychotic reactions, generally lasting but a few hours, but occasionally as long as a week; the reaction seemed dose-related and its main features included paranoid ideation, illusions, hallucinations, delusions, depersonalization, confusion, restlessness, and excitement. There can be delirium, disorientation, and marked clouding of consciousness*” (33). Numerous case reports have since then documented the acute psychotomimetic symptoms of cannabis intoxication, including depersonalization, derealization, paranoia, ideas of reference, flight of ideas, pressured thought, disorganized thinking, persecutory delusions, grandiose delusions, auditory/visual hallucinations, and impairments in attention and memory (30, 33–42) in about 20–50% of individuals (43, 44).

In a survey of ultra-high-risk and recent-onset patients with psychosis (45), 37% of subjects reported that their first psychotic symptoms appeared during cannabis intoxication. The subjects also reported feeling more anxiety, depression, and suspiciousness immediately after cannabis use than cannabis-using controls. Another recent study of first-episode psychosis (FEP) patients (*n* = 109) found that daily cannabis users were significantly more likely to have an acute onset of psychosis than non-daily users (46). Evidence from case reports and surveys is limited, however, by confounds such as observer bias, effects of other illicit drugs, and failure to exclude negative and cognitive symptoms prior to onset of positive symptoms.

#### Evidence from anecdotal reports and surveys of the effects of medicinal cannabinoids

With the pioneering work of Mechoulam in 1964, the individual constituents of cannabis were characterized (47). The identification of THC as the main psychoactive agent led to the synthesis of dronabinol (synthetic THC) and other non-psychotropic synthetic cannabinoids such as levonantradol and nabilone (9-trans-ketocannabinoid), which were thought to have specific antiemetic, analgesic, and antispastic effects. The use of these agents for the treatment of pain syndromes, chemotherapy-induced nausea, and spasticity in multiple sclerosis was followed by reports of transient psychotomimetic effects among patients. The psychotomimetic effects reported were similar to that with cannabis including “loss of control,” thought disturbances, feelings of unreality, apprehension, fear and paranoia, anxiety and panic, dissociation, depersonalization, dysphoria, difficulty concentrating, hallucinations, perceptual alterations, amnesia, and anxiety (48–62). These effects were dose-related and proportional to the affinity of the compound for the CB_1_R. The high incidence of intolerable behavioral side effects in fact, led to the discontinuation of drug development of levonantradol as an analgesic. In a systematic review of 30 studies that examined the efficacy of dranabinol, nabilone, or levonantradol for chemotherapy-induced nausea and vomiting Machado Rocha et al. (63) found that synthetic cannabinoids was responsible for 30% of dropouts; with 6% patients developing hallucinations and 5% developing paranoia. In another systematic review, Tramer et al. (64) found that patients receiving synthetic cannabinoids had a higher relative risk of developing dysphoria or depression [RR 8.06 (95% CI 3.38–19.2)], hallucinations [RR 6.10 (95% CI 2.41–15.4)], and paranoia [RR 8.58 (95% CI 6.38–11.5)] than those receiving non-cannabinoid antiemetics. Importantly, hallucinations and paranoia were seen exclusively with cannabinoids, and not with other antiemetic agents; and these effects appeared to be related to dose, potency, and frequency of administration.

#### Evidence from anecdotal reports and surveys of the effects of synthetic cannabinoids (Spice, K2)

The emergence of potent synthetic cannabinoids as drugs of abuse in the last decade provide another source of evidence pointing to the link between cannabinoids and psychosis (8). These compounds, collectively referred to as Spice or K2, comprise a mixture of synthetic cannabinoids such as CP-47,497, CP-47,497-C8, JWH-018, JWH-073, JWH-081, JWH-122, JWH-210, JWH-250, HU-211, and RCS-4 (65–72). It should be noted that, unlike THC, which is a weak partial agonist of brain CB_1_Rs, the synthetic cannabinoids are highly potent, full agonists of CB_1_R, which would predict more robust effects. Spice has gained popularity as a drug of abuse since it is more psychoactive than cannabis, is readily available over the Internet (advertised as “natural herbs” or “harmless incense” under brand names such as Spice, K2, Yucatan Fire, Skunk, Moon Rocks), and is non-detectable in standard urine toxicological tests. In some countries, including much of the United States and Canada, synthetic cannabinoids are available at gas stations and head-shops as natural herbs and incense; this contributes to its perception as safe and legal among users.

There are no controlled-studies on the psychotomimetic effects of synthetic cannabinoids (73); available information about their effects in humans consists of retrospective case reports from emergency room (ER) visits (69, 70, 74), surveys (12–14), reports from the American Association of Poison Control Centers (AAPCC) (75), and from media and law-enforcement agencies on catastrophic events related to their use (76–81). There has been a substantial increase in ER visits resulting from acute behavioral effects following use of these synthetic cannabinoids. The psychotomimetic effects reported include anxiety, agitation, disorientation, hallucinations, and paranoia (69, 70, 82–84). In an Internet survey, Spice/K2 users most commonly endorsed feeling paranoid (11%), hallucinating (3%), and feeling as if in a dream-like state (26%) “most of the time” or “every time” they used “Spice” (14). The AAPCC reported an exponential increase in call volume related to the use of Spice/K2 from 53 calls in 2009 to over 6000 in 2011 with callers reporting symptoms of agitation, drowsiness, and hallucinations (62% of calls) (75).

Case reports document the ability of these compounds to precipitate a psychotic relapse in patients with pre-existing psychotic disorders and psychotic symptoms in those with no prior history of psychosis (12, 74, 85). Müller et al. (86) reported on a 25-year-old man with a history of psychotic episodes precipitated by cannabis use and a family history of schizophrenia who had been stable for 2 years and had a psychotic relapse comprising anxiety, paranoid delusions, and hallucinations after smoking Spice on three occasions in 1 month. Every-Palmer described sudden agitation, disorganization, and delusions in five forensic patient who had consumed Spice containing JWH-018 and/or CP-47,497 (85). Of the five patients, only one retained insight into the possible psychotogenic nature of “Spice” (85). In a follow up survey of 15 inpatients with serious mental illness in a forensic psychiatric facility, Every-Palmer reported that patients commonly experienced anxiety and psychotomimetic effects, few developed tolerance, and none reported withdrawal symptoms (12).

Psychotic symptoms are also reported in patients with no previous history of psychosis. The adverse clinical events documented in case reports include altered consciousness, confusion, anxiety, irritability, agitation, paranoia, hallucinations, and psychosis (70, 82, 85–87). However, the majority of case reports to date discuss people 25 years or younger (84, 88). Therefore, it is possible that “Spice” exacerbates a pre-existing prodromal syndrome. Case reports and cross-sectional surveys are only able to show an association and cannot elucidate causation.

The sparse literature on Spice/K2 effects reviewed above has a number of limitations, including selection bias, reliance on the accuracy of written record or subject recall, uncontrolled nature of the evidence, the inadequate characterization of cases, lack of standardized assessments, confounding effects of concomitant drug use, different doses and routes of administration, and variable individual expectancy, set, and setting. Cases reported by the media and law-enforcement may represent extremes that might not be generalizable. The temporal profile, range, and intensity of Spice/K2 effects, and whether the effects are dose-related or biphasic, are not known. Furthermore, the relationship between dose, effects, and blood/urine levels of the parent compound and metabolites is not known.

## Immediate Effects of Cannabinoids: Experimental Evidence

Experimental studies provide an opportunity to control variables such as dose, route of administration, and setting, while employing a randomized-control paradigm. Studies have variously examined the effects of smoked cannabis, cannabis extract, oral, and intravenous THC and CBD on positive psychotomimetic symptoms, negative symptoms, cognitive, and psychophysiological measures. Although, early semi-experimental studies of cannabis in the early 1900s using oral cannabis or cannabis extract [reviewed in Ref. (18)] demonstrated cognitive and perceptual effects of cannabis, D’Souza et al. (89) were the first to characterize the profile of positive psychotomimetic symptoms, negative symptoms, and cognitive effects of intravenous THC in healthy individuals. Despite varying routes of administration, experimental studies have yielded some consistent results regarding the effects of cannabis, THC, and CBD. There have, however, not been any controlled experimental studies of the synthetic cannabinoids in humans to date.

In the following sections, we provide a brief summary of the consistent effects noted with cannabis, THC, and CBD. Interestingly, cannabis and THC produce the full range of positive psychotomimetic symptoms, negative symptoms, and cognitive deficits seen in schizophrenia, while CBD has been shown to have anxiolytic properties and even inhibit the psychotomimetic effects of THC (90–92).

### Positive symptoms

**Figure 1:**
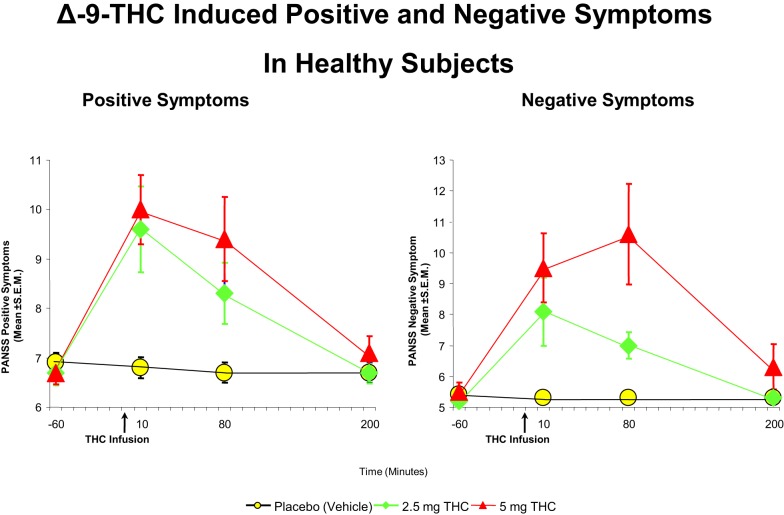
**Effects of THC on the seven-item positive symptom and negative symptoms subscales of the Positive and Negative Syndrome Scale (PANSS)**. THC at both a low dose (2.5 mg) (green) and moderate dose (5 mg) (100) induce an increase in positive and negative symptoms, compared to placebo (yellow). Adapted from Ref. (89).

**Figure 2:**
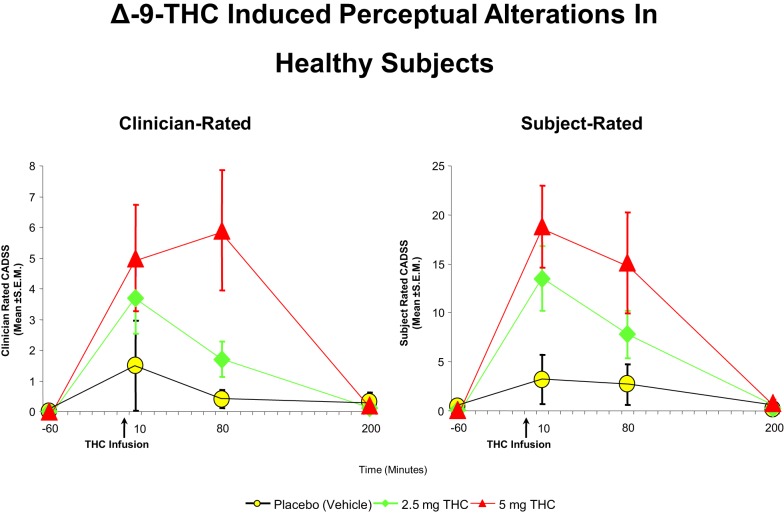
**Effects of THC on the clinician- and subject-rated subscales of the Clinician Administered Dissociative Symptoms Scale (CADSS), a measure of perceptual alterations**. THC at both a low dose (2.5 mg) (green) and moderate dose (5 mg) (100) induce an increase in perceptual alterations as rated by the clinician and the subject, compared to placebo (yellow). Adapted from Ref. (89).

Cannabis extract containing predetermined quantities of THC (93, 94) and THC alone (32, 73, 89, 92, 94–99) have been shown to produce a range of transient, positive symptoms, that are qualitatively similar to the positive symptoms of schizophrenia. These symptoms include suspiciousness, paranoid and grandiose delusions, conceptual disorganization, fragmented thinking, and perceptual alterations. Additionally, cannabis and THC also result in depersonalization, derealization, alterations in sensory perception, and feelings of unreality. These effects have consistently been demonstrated with smoked cannabis, oral cannabis extract/THC (dose range 5–20 mg), intravenous THC (dose range 0.015–0.03 mg/kg), and intrapulmonary administration via a vaporizer (32, 73, 89, 92, 94–99). In the first study of its kind in a carefully controlled laboratory setting, D’Souza et al. (89), administered intravenous THC in two doses (2.5 and 5 mg), in a double-blind, randomized, placebo-controlled study in healthy adults (*n* = 22). Subjects were screened to rule out significant psychiatric disorder or family history of Axis I disorders (89). The study found that THC produced transient positive psychotic symptoms (Figure 1) including perceptual alterations, negative symptoms, mood symptoms such as euphoria and anxiety, and also cognitive deficits, especially in attention, working memory, and verbal recall (Figure 2). In a similar study in healthy individuals, using almost identical methods except for a lower dose of THC, Morrison et al. (95) showed that intravenous THC (2.5 mg) produced similar effects on positive psychotic symptoms, mood, and cognition.

The effects of dopamine D_2_-receptor antagonists on the psychotomimetic effects of THC are not clear. For example, in some studies, olanzapine (101) and haloperidol (102) were shown to attenuate the psychotomimetic effects of THC. However, D’Souza et al. showed that acute treatment with haloperidol did not attenuate the psychotomimetic effects of THC in healthy subjects (103) and chronic antipsychotic treatment failed to protect schizophrenia patients from the symptom exacerbating effects of THC (104). The potential antipsychotic and anxiolytic effects of CBD have drawn increasing attention. In a functional magnetic resonance (fMRI) study of brain responses to emotional expression of faces, Fusar-Poli et al. (90) found that while THC resulted in increased psychotic symptoms and increased skin conductance responses during processing of fearful faces; CBD, on the other hand led to a reduction in anxiety and a decrease in skin conductance response. A separate fMRI study showed that THC and CBD had opposite effects on blood oxygen-level dependent (BOLD) responses in tasks of verbal recall, response-inhibition, processing fearful facial expressions, auditory processing, and visual processing (91). Some limitations notwithstanding, this study provided some important leads into the differential effects of CBD and THC.

Time perception abnormalities are known to occur in schizophrenia, but have received little attention (105–108). Cannabinoids have been shown to alter time perception in both preclinical (109–112) and clinical studies (113–117). In the largest double-blind, randomized, cross-over, placebo-controlled study to date, Sewell et al., showed that THC at different doses induced time overestimation and underproduction compared with placebo (118). Cannabinoids have also been found to disrupt performance on visual information processing in the binocular depth inversion task, a potential surrogate marker for psychosis seen in patients with acute paranoid schizophrenic or schizophreniform psychosis (119). This effect has been observed with cannabis resin (120), nabilone (a synthetic analog of THC) (121), dronabinol (a synthetic isomer of THC) (119), and in chronic cannabis users (122).

### Negative symptoms

Delta-9-tetrahydrocannabinol also produces a range of effects similar to the negative symptoms of schizophrenia, including blunted affect, emotional withdrawal, psychomotor retardation, lack of spontaneity, and reduced rapport (89, 97). It is difficult to determine whether these “negative symptoms” were primary or were a consequence of the sedating and cataleptic effects of cannabinoids observed in animal studies. Morrison et al. (97) however, showed that the effect of THC on negative symptoms was independent of effects on sedation. It is also unclear if the negative symptoms were a manifestation of internal preoccupation with positive psychotic experiences. Furthermore, acute pharmacological studies may be limited in their capacity to model negative symptoms.

### Cognitive deficits

Cannabis, THC and other synthetic cannabinoids also produce transient, dose-related cognitive impairments, especially in the domains of verbal learning, short-term memory, working memory, executive function, abstract ability, decision-making, and attention (123–129). These effects are not limited to humans but are also seen in rodents and non-human primates [reviewed in Ref. (130, 131)]. Interestingly, the profile of impairment observed in different cognitive domains is similar to that observed in schizophrenia (132).

**Figure 3:**
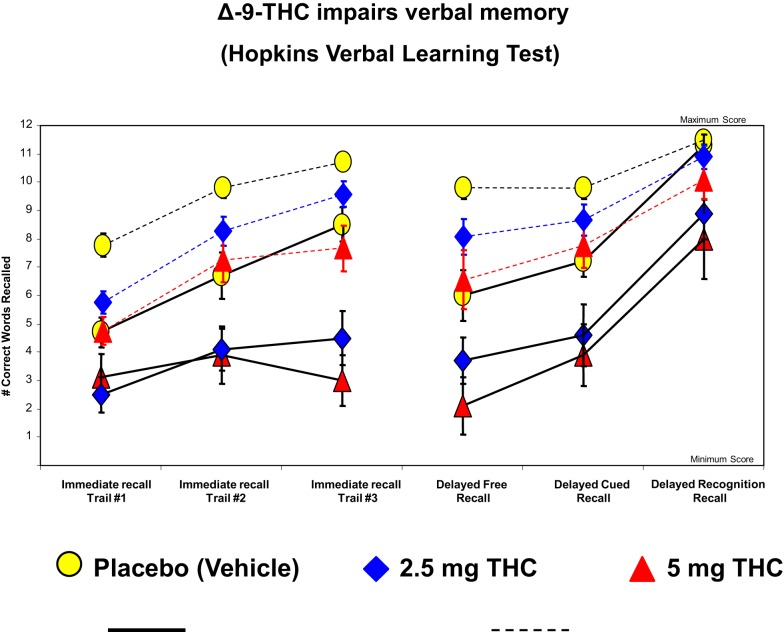
**Effects of THC on the immediate free recall, delayed free recall, delayed cued, and recognition recall measured by a 12-word learning task (Hopkins Verbal Learning Test), a measure of verbal memory**. THC at both a low dose (2.5 mg) (blue) and moderate dose (5 mg) (100) induce an immediate free recall, delayed free recall, delayed cued, and recognition in patients with schizophrenia (solid line) and healthy individuals (dotted line), compared to placebo (yellow). Adapted from Ref. (89).

The cognitive impairment produced by THC is most pronounced in the domain of verbal learning and memory (129), which is also one of the domains of significant impairment in schizophrenia (132). Figure 3 illustrates the effects of THC on the Hopkins Verbal Learning Test (HVLT) in healthy subjects (104). THC has been shown to produce robust dose-dependent impairments on both immediate and delayed (30 mins) verbal recall. THC also increased the number of “false positives” and “intrusions” on the HVLT. Similar findings have been recently reported by Henquet et al. (133) and Morrison et al. (95).

The acute effects of cannabinoids are likely modulated by genetic and personality factors. This would explain why only a small minority of people experience the psychotomimetic effects of cannabinoids. Henquet and colleagues examined the effects of the interaction of Catechol-*O*-methyl transferase (COMT) polymorphism and a trait index of psychosis liability on smoked THC (0.3 mg/kg) on cognitive performance and psychosis in 30 healthy individuals (133). They found that individuals with the Val/Val polymorphism and high scores on psychosis liability had higher THC-induced psychotic symptoms.

### Psychophysiological effects

Psychophysiological effects refer to measures that attempt to examine the physiological basis of psychological processes. In the study of cannabinoids, these effects have primarily been demonstrated using electroencephalography (EEG). EEG measures of information processing, such as event-related potentials (ERPs) and neural oscillations, offer a more proximal index of neural events in humans with exquisite temporal precision (134). ERPs are averaged EEG responses time-locked to particular stimuli or events. ERPs relevant to psychosis include: (1) P50 – a measure of auditory sensory gating, (2) P300b – a measure of directed attention, contextual updating of working memory, and the attribution of salience to deviant or novel stimuli (135), (3) P300a – a measure of novelty detection, and (4) mismatch negativity (MMN) – a measure of processing and memory of deviant stimuli. These ERP measures have been reported to be abnormal in schizophrenia and have been considered biomarkers of the disorder. Abnormalities in neural oscillations have also been noted in schizophrenia and in chronic cannabis users.

Deficits in auditory sensory gating, as demonstrated by a disruption in P50 response, have been shown in patients with schizophrenia (136–140). The cannabinoid agonists CP-55940 and WIN 55,212-2 have been shown to disrupt sensory gating in rats (141, 142). However, there are no studies that have examined the acute effects of cannabinoids on sensory gating (P50) in humans. In contrast, there are cross-sectional studies comparing heavy, chronic cannabis users to healthy controls that have shown that chronic cannabis users show disruptions in P50 suppression (143, 144), which was evident despite subjects abstaining for 24 h. These findings suggest that chronic cannabis use is associated with disruption in sensory gating. Furthermore, the degree of disruption in sensory gating was found to correlate positively with the magnitude of cannabis exposure (138, 145), suggesting a dose-response relationship.

The P300 is a late positive, post-attentional ERP component thought to be related to directed attention, contextual updating of working memory, and the attribution of salience to deviant or novel stimuli (135). Deficits in P300 amplitude and latency have been demonstrated in patients with schizophrenia (136, 139, 146–152). THC has been shown to cause a reduction in the amplitude of the P300 response in several paradigms such as a visuospatial N-back working memory task (153), and auditory choice reaction task (154, 155). D’Souza et al. examined the effect of several doses of intravenous THC on the P300 response in healthy individuals and showed that THC decreased the amplitude of both the novelty P300a and target P300b (155), while also producing concomitant psychotomimetic effects. There was no impairment in the latency of the P300 response or in the N100 response, indicating that THC disrupted cortical processes responsible for context updating (P300b) and the automatic orientation of attention (P300a), without affecting early sensory registration (N100) or processing speed. Studies of chronic cannabis users have however been equivocal, with studies variably showing decreased P300 amplitudes (156), increased P300 latency (157), increased P300 amplitude (157, 158), or shorter P300 latency (159). Although the reasons behind these discrepant results are unclear, it is possible that chronic cannabis users are impaired during more cognitively challenging selective attention tasks (156, 157, 160), but retain normal ERP responses during simple dual-stimulus discrimination tasks (158, 159).

Mismatch negativity is an automatic, pre-attentive, and negative-voltage ERP component that occurs ~100–200 ms after a deviant auditory stimulus that differs in frequency or duration from a sequence of standard auditory stimuli. It is thought to represent basic auditory information processing, and sensory memory generated primarily in the superior temporal and prefrontal cortex (PFC), while being relatively independent of attention (161, 162). Deficits in MMN have been shown in patients with schizophrenia, early psychosis, and high-risk subjects (163, 164). While oral THC did not produce any acute changes in MMN amplitude (93), studies in chronic cannabis users have demonstrated decreased MMN amplitudes in the frequency deviance condition (154, 165–167). There also appears to be a dose–response effect in the MMN response with long-term and heavier users of cannabis demonstrating significantly lower MMN amplitudes compared to short-term or light users and duration of cannabis exposure showing a negative correlation with MMN amplitudes (154, 165).

Cannabinoids have been shown to disrupt theta band (4–8 Hz) neural oscillations in rats (168). Similar disruption in theta band power was demonstrated following smoked cannabis (169). The degree of disruption in theta band power correlated with deficits in working memory performance in this study. Studies of neural oscillations in chronic cannabis users have demonstrated attenuation of high frequency activity in the beta range (13–29 Hz) (145, 170) and in the gamma range (30–50 Hz) (145, 171). These findings are very interesting in light of accumulating evidence that schizophrenia may be primarily a disorder of abnormal neural oscillations and synchrony [reviewed in Ref. (172)] and that neural oscillations may also be important in the organization of the networks in the brain (173).

## Acute Psychosis Outlasting Intoxication

The use of cannabinoids are also associated with acute *episodes* of psychosis that: (1) manifest immediately following exposure, (2) last beyond the period of intoxication, and (3) sometimes require clinical intervention. This is distinct from the effects previously described, which do not outlast the period of intoxication. Most of the literature about this phenomenon comes from small case series and case reports. The phenomenology, duration, and course of such cases – which we refer to as cannabis-induced acute and persistent psychosis (CIAPP) – have not been systematically characterized.

In the 1890s, the Indian Hemp Drugs Commission undertook a study to examine the effects of cannabis use. The commission reported that “excessive” cannabis use was responsible for psychotic reactions in 9.5% (222/2344) of cases in asylums in India. Chopra et al. reported a series of patients admitted to a psychiatric hospital in India for cannabis related psychosis (29, 30). The psychosis was typically preceded by ingestion of large doses of cannabis and was characterized by hallucinations, delusions, paranoia, depersonalization, amnesia, emotional lability, confusion, and disorientation. Similar case series have been reported from other geographical areas including Sweden, Denmark, the Caribbean, Scotland, UK, USA, and South Africa (37, 174–181). These case reports suggest that when cannabis use is stopped, the acute psychotic episodes resolve (quicker in comparison with “endogenous” psychoses) (37, 39, 177, 178, 180, 182–186), and do not recur unless cannabis use resumes [reviewed in Ref. (187)]. However, since follow up was only for a few months, the long-term course and outcome, the clinical implications, and prognostic significance of these cases remains unclear. Several recent large (*n* = ~20,000) studies suggest that, over long-term (~8 year) follow up, ~50% of patients without any pre-existing psychiatric disorder who were hospitalized for cannabis-induced psychosis, were later re-diagnosed with a schizophrenia-spectrum disorder (181, 188); that number increased to ~75% when the diagnosis included *any* psychotic outcome (181). These observations suggest that hospitalization for CIAPP may be a harbinger of a recurrent psychotic disorder that we currently classify as schizophrenia. More recent case reports and retrospective studies continue to demonstrate the close temporal relationship between use of cannabis and the onset of a psychotic disorder, sometimes quite indistinguishable from schizophrenia (189, 190). In fact, the International Classification of Diseases-10 (ICD-10) allows for the psychotic effects of cannabis to be coded as both an acute polymorphic psychotic disorder and a protracted substance-induced psychotic disorder. It is conceivable that, as suggested by Rounsaville (191), these cases may actually represent a distinct persistent psychotic disorder.

## Delayed and Persistent Effects of Cannabinoids

The evidence for persistent effects of cannabinoids in humans comes from large-scale epidemiological studies and from studies in chronic cannabis users. In the following section, we examine the evidence linking cannabis use and persistent psychotic disorder, including negative and cognitive symptoms.

### Persistent psychotic disorder

The evidence for the association between cannabis use and persistent psychosis comes from both cross-sectional studies (192–196) and longitudinal epidemiological studies, including the Swedish military conscript cohort (197–199), the Netherlands Mental Health Survey and Incidence Study (NEMESIS) (20), the German prospective Early Developmental Stages of Psychopathology Study (EDSP) (24), the Dunedin cohort (19, 200), and the Christchurch Health and Development Study (CHDS) birth cohort (23).

The first study to draw attention to the association between cannabis use and psychosis was the Swedish conscript study (197), in which Andreasson et al. followed a cohort of 45,570 Swedish military conscripts (97% of all Swedish males aged 18–20 years) from 1969 to 1970. The investigators observed a dose–response relationship between cannabis use (via self report) at initiation of military service and hospitalization for a psychotic disorder over the ensuing 15 years, with maximal risk among those who had smoked cannabis more than 50 times. Conscripts who reported having used cannabis at least once in their lifetime had a 2.4-fold (95% confidence interval 1.8–3.3) increased risk of developing schizophrenia over the course of 15 years. This relative risk rose to sixfold (95% CI 4–8.9) in those who had used cannabis more than 50 times in their lifetime. The risk remained significantly high despite adjusting for other factors such as psychiatric illness at the time of conscription, solvent abuse, and parental separation. In a 27-year follow up study of the same cohort and a re-analysis of the data, Zammit et al. replicated the findings of Andreasson et al., showing that cannabis use was associated with a linear increase in the risk of developing schizophrenia; the relative risk increasing from 2.2 (95% CI 1.7–2.8) in those who had used cannabis at least once, to 6.7 (95% CI 4.5–10) in those who had used cannabis more than 50 times in their lifetime (198). When potential confounders such as IQ sore, disturbed behavior in childhood, psychiatric diagnosis at conscription, cigarette smoking, degree of social integration, and place of upbringing were included in the regression analysis, the adjusted relative risk was 1.5 (95% CI 1.1–2.0) in those who had used cannabis at least once and 3.1 (95% CI 1.7–5.5) in those who had used cannabis more than 50 times in their lifetime. The relative risk for schizophrenia was significantly higher in those who developed schizophrenia within 5 years of conscription, which raises questions about the direction of causality. In other words, this preliminary analysis could not distinguish whether cannabis use led to schizophrenia or whether subjects used cannabis in an attempt to self-medicate incipient symptoms of schizophrenia. In a secondary analysis that excluded those who developed a diagnosis of schizophrenia within 5 years of conscription, the adjusted relative risk remained significant only for those who had used cannabis more than 50 times (adjusted relative risk = 2.5, 95% CI 1.2–5.1). The study needs to be interpreted with caution: while 24.3% of the sample had used any drug, a very small percent (3.4%) had used only cannabis. While the analysis controlled for cigarette smoking, it failed to control for the use of stimulants and other drugs. Also, the fact that presumably weak confounders (such as “place of upbringing” and “cigarette smoking”) contributed substantially, along with other variables in reducing the adjusted relative risk by ~50% in the regression analysis highlights the difficulties inherent in interpreting epidemiological data and raises the issue of other unknown confounders. Similar criticisms of the studies from the Swedish conscript cohort have been raised by other authors (201–203), including the facts that: (a) the use of other drugs was more common in the cannabis-using group, (b) the association between cannabis use and schizophrenia may be mediated by a third, as yet unknown factor, and (c) the follow up study, a quarter century later, failed to address the issue of confounding due to use of other drugs, many of which are also known to precipitate psychosis.

Using the NEMESIS cohort, van Os et al. reported that cannabis use at baseline was associated with an increased risk of psychosis (20). The study assessed 7076 subjects at baseline (1996), 5618 subjects at a first time-point (1997), and 4848 subjects at a second time-point (1999) via telephonic interviews, and found 10 subjects who developed psychosis, while 38 subjects endorsed individual items on the Brief Psychiatric Rating Scale (BPRS). The findings of the study are limited by the small numbers in the outcome of interest (25) despite the large sample size.

The EDSP study, which used in-person interviews in the assessment of 923 individuals from the general population (aged 14–24 years), showed that cannabis use was associated with an increased risk of psychotic symptoms and persistent use increased this risk further (28). Importantly, this study yields evidence for a unidirectional relationship between cannabis use and psychosis. This is in contrast with another recent study (22), which showed the relationship to be bi-directional, alluding to the possibility of a phenomenon of “self-medication,” a topic that is further discussed below.

The Dunedin cohort study (19) examined data from 759 subjects of the population birth cohort comprising 1037 individuals born in Dunedin, New Zealand, in 1972–1973. The study collected information on psychotic symptoms at age 11, drug use at ages 15 and 18 years, and assessed psychiatric symptoms at age 26. Cannabis use by age 15 and 18 years was found to be associated with more schizophrenia symptoms at age 26 years; and the association remained significant despite controlling for the presence of psychotic symptoms at age 11 years. The association was also found to be stronger with earlier use. Those who used cannabis by age 15 years were also four times more likely to have a diagnosis of schizophreniform disorder; the risk was reduced by 31% and no longer significant after controlling for psychotic symptoms at age 11 years, pointing to the possibility of reverse-causality.

Fergusson et al. attempted to validate a possible causal link between cannabis use and psychosis in a dataset of a 25-year longitudinal study in New Zealand (the CHDS birth cohort comprising 1265 children) (23). The study showed that daily use of cannabis was associated with 2.3- to 3.3-fold higher risk of psychosis than among non-users. One of the limitations of the study is that the data was derived from 10 items of the Symptom Checklist-90, the items on which overlap with personality traits such as schizotypy and paranoia and that the study did not attempt to delineate psychotic symptoms due to the acute effects of cannabis use from persistent effects (204).

This finding of increased psychosis risk has been reported in several other prospective studies (19–21, 24). The cumulative evidence for the association between cannabis and psychosis have been examined in five systematic reviews (25, 205–208), four of which (25, 205, 207, 208) found a consistent association between cannabis use and psychosis. The review by Macleod et al. (206) did not find a consistent association, but has been critiqued for failure to perform a meta-analysis. The inconsistent results of the systematic reviews are also likely due to different inclusion/exclusion criteria, different methodology, and different outcome measures (209). In the latest systematic review by Moore et al., any cannabis use (pooled adjusted OR = 1.41, 95% CI 1.20–1.65) was associated with a 40% increased risk of psychotic disorder, and the risk increased in a dose-dependent fashion with greater cannabis exposure (OR = 2.09, 95% CI 1.54–2.84) (25).

While the evidence supporting an association between cannabis exposure in adolescence and later psychosis is largely consistent, the evidence has been challenged on many counts (210), including sampling bias; under-powered sample sizes; presence of unknown confounders; difficulty distinguishing psychotic symptoms from psychotic disorder in longitudinal studies; direction of causality; lifetime exposure to multiple drugs; and period-, time-, and cohort-effects.

### Negative symptoms

Chronic and heavy cannabis use has been associated with a syndrome characterized by a predominance of negative symptoms, referred to as an “amotivational syndrome” (175, 187, 211–213). The features of this syndrome include apathy, amotivation, social withdrawal, narrowing of one’s personal repertoire of interests, lethargy, impairment in memory and concentration, impaired judgment and decision-making, and poor socio-occupational functioning. All these symptoms share similarities with the negative symptoms of schizophrenia. The nosological status of the syndrome is, however, debated. Further, the confounding effects of concomitant poly-substance abuse, poverty, low socio-economic status, or pre-existing psychiatric disorders may explain the association (214, 215).

This literature is in contrast with the finding that healthy, cannabis users have lower scores on negative schizotypy compared to healthy, drug-free individuals (158, 216), and that patients with schizophrenia who use cannabis have less negative symptoms compared to those who do not use cannabis (217, 218). The cross-sectional nature of these studies and lack of information regarding scores at baseline makes it difficult to conclude if cannabis does not indeed cause a worsening of negative symptoms compared to baseline.

### Cognitive deficits

Several studies suggest that chronic, heavy cannabis use leads to impairments in memory, attention, working memory, executive function and IQ (219–227). Solowij and Mitchie suggested that cognitive dysfunction associated with long-term or heavy cannabis use is a cognitive endophenotype of schizophrenia (139). In a comprehensive review, Solowij and Battisti concluded that chronic heavy cannabis use was associated with impairments in memory (224) that persisted beyond the period of acute intoxication and was related to the frequency, duration, dose, and age of onset of cannabis use. Fontes et al. evaluated the neuropsychological performance of 104 chronic, heavy cannabis users and found that, compared to controls, chronic cannabis users had significant impairment on the cognitive domains of sustained attention, impulse control, and executive functioning (226). Additionally, similar to the literature on the risk of psychosis, individuals who used cannabis in adolescence (before the age of 15 years) had greater deficits. The authors however, did not assess whether subjects were in withdrawal or had residual effects from their last use of cannabis at the time of assessment.

While chronic, heavy cannabis users have deficits in cognitive processes, especially memory and attention in the context of ongoing cannabis use, the question of whether these impairments are persistent or a result of withdrawal and residual effects is unclear. While one study demonstrated an absence of persistent neuropsychological deficits in frequent long-term cannabis users after 28 days of abstinence (228), other studies have shown variable durations to full recovery, ranging from a week (229), to 28 days (221), to 3 months of abstinence (230), with some studies showing recovery only after an average of 2 years of abstinence (187, 231). A recent review provides a summary of the literature to date (225). Among studies in which neuropsychological assessments were performed 3 weeks or later after last use of cannabis, five out of seven studies showed no impairment in attention (221, 228, 232–236), while two showed persisting impairment (222, 231). One study revealed a trend toward impairment in decision-making/risk-taking (237). There was no impairment on response-inhibition measured by the Stroop test (221, 222, 233–235), and on working memory (236) while all (221, 222, 233, 234) but one (235) found an impairment on the Wisconsin Card Sorting Test, a test of set shifting. There was no impairment in verbal memory in two (228, 233) of the three studies that used the Buschke’s Selective Reminding Test (BSRT), a test of memory of word lists. When the data from the third study (234) was stratified based on age at onset of cannabis use, significantly greater impairment was noticed in those who had first use cannabis before the age of 17 years, suggesting that, as for positive symptoms, earlier age of onset of cannabis use may be associated with greater persistent cognitive deficits. It is important to note that none of these studies were designed to determine whether the cognitive impairments predated cannabis use.

Previous cross-sectional experiments have reported inconsistent results with some suggesting that chronic cannabis use impairs performance on tests of intelligence (238, 239), while others finding no impairment (240, 241). A recent longitudinal study examined 1037 subjects followed from birth to age 38 years (242). Cannabis use was evaluated at ages 18, 21, 26, 32, and 38 years while neuropsychological testing was conducted at ages 13 and 38 years. The experiment determined that those who persistently use cannabis are more likely than non-users to experience a significant decline in IQ. The findings persisted even after controlling for level of education and impaired IQ was found to be particularly true for the subjects who began to use cannabis during adolescence as opposed to during adulthood. Those who began to use cannabis during adolescence exhibited an eight-point decrease in IQ between childhood and adulthood. Another important finding of the study was that the decline in IQ did not appear to reverse after cannabis use ceased (242).

Some studies that have examined cognitive performance among patients with schizophrenia have made a case that patients with schizophrenia and comorbid cannabis abuse have better cognitive performance than patients without comorbid cannabis abuse (243–246). Emerging evidence, however, suggests that patients with cannabis use have higher premorbid IQ (247). The findings are not inconsistent with the experimental data; it is likely that persons who smoke cannabis have higher premorbid IQ, as evidenced by their ability to procure an illegal substance while evading the law. Therefore, although continued cannabis use results in a decline in their individual cognitive performance (242, 248, 249), when compared to non-users they appear to have relatively better cognitive performance. Furthermore, abstinence from cannabis may be associated with better cognitive performance among male patients with schizophrenia (248).

## Moderators/Mediators of the Link between Cannabis and Psychosis

### Age of exposure

Epidemiological evidence suggest that the earlier the age of exposure to cannabis, the greater the risk of a psychosis outcome (19). Dragt et al. showed that younger age of onset of cannabis use is associated with earlier symptoms of anxiety, social withdrawal, derealization, memory impairment, and difficulties in concentration, with effects being more pronounced in patients with heavier cannabis use (250). Another recent study found that early onset cannabis use was only associated with earlier onset of psychosis when cannabis use began by age 14 (251). A large meta-analysis of 83 studies found that the age of onset of psychosis in cannabis users was 2.7 years younger than in non-users (252). Animal studies have shown that exposure to cannabinoids in adolescence has more deleterious effects than exposure in adulthood (253–257).

It is being increasingly recognized that adolescence may be a particularly critical period of increased vulnerability to the effects of cannabis. Additionally, factors such as schizotypy, other trait measures of liability to psychosis, and childhood abuse may moderate the risk of schizophrenia with prolonged and persistent cannabis use. As discussed above, the 26-year longitudinal study of the Dunedin cohort showed that earlier cannabis use is associated with a greater risk of psychotic disorder. However, when adjusted for psychotic symptoms at age 11, the association between cannabis use and subsequent psychotic disorder was no longer significant but remained elevated (OR = 3.1) (19). The small sample size may limit the interpretation of these results.

These studies suggest a “window of vulnerability” hypothesis: a critical period during early adolescence where the brain is particularly susceptible to the psychosis-inducing effects of cannabis (19, 250, 251, 253–258). One possible explanation for the “window of vulnerability” theory is that cannabis may affect the brain during a critical period of development and maturation. Brain development and maturation processes – including neuronal migration and differentiation, synaptogenesis, axon formation, and dendritic proliferation, myelination, pruning, apoptosis, and activity-dependent changes – begin *in utero* but continue into the early 20s or even later (259–264). Cannabis may disrupt one or more of these processes.

A retrospective study of 997 subjects by Stefanis and colleagues showed that, after adjusting for family history, there was a consistent relationship between the age of cannabis initiation and FEP, with an average time of 7–8 years (265). This finding does not support the “window of vulnerability” hypothesis, but rather indicates that the brain (at least in years 12–19) is continually sensitive to cannabis.

The association between age of onset of cannabis use and worse outcomes could simply reflect that earlier use is more likely to become longstanding, thus increasing the overall exposure to cannabis. An alternate explanation for the association between age of exposure to cannabis and psychosis is that those prone to early psychosis may “self-medicate” with cannabis to relieve symptoms (22, 266). However, this has not been supported by recent literature (28, 250, 251, 267). These studies are limited in that they have relied on measuring only positive psychotic symptoms as an indication of psychosis onset, although it is known that negative symptoms and cognitive deficits predate the onset of positive symptoms (268) and even predict conversion to psychosis in high-risk individuals (269). The interpretation of the data is also limited by the fact that cannabis use at an early age may be part of a broader pattern of externalizing behavior in response to difficult family circumstances (270, 271). Children and adolescents who begin cannabis use at an earlier age may represent a distinct sub-population that differs in ways that have not been accounted for (such as history of abuse or family socio-economic level) in the aforementioned studies.

### Family history

Early studies have indicated that a positive family history of schizophrenia may increase risk for cannabis-induced psychotic disorders. One such study found that among patients admitted for acute psychosis, those who tested positive for cannabinoids in urine toxicology screens were 10 times more likely (7.1 vs. 0.7%) to have a positive family history for schizophrenia than patients without a positive urine toxicology screen (272). This finding implicated a familial predisposition to persistent psychotic disorders precipitated by cannabis use. Thus, in a genetically predisposed sub-population, cannabis confers a marked risk for psychosis. Most studies since have confirmed an association between a family history of psychotic disorder and an increased risk of cannabis-induced psychosis, though the association is more modest than the original study. Bersani et al. found that among schizophrenia patients, 24% of cannabis users had a positive family history of psychotic disorder vs. 10% (217). The largest study to investigate this association (*n* = 2,276,309) found a 2.5-fold increased risk of developing cannabis-induced psychosis in children of mothers with schizophrenia but no increased risk of conversion to schizophrenia (273). Further studies that have followed patients over time have shown that among patients who are admitted with an initial diagnosis of cannabis-induced psychosis, almost 50% convert to schizophrenia or some other psychotic disorder (181, 188). Boydell et al., found, in a retrospective study of 757 first-episode schizophrenia patients (24% who used cannabis in the year prior to presentation), that among patients with schizophrenia, cannabis users did not differ significantly from those not using cannabis in terms of a positive family history of schizophrenia (15 vs. 12%) (274). More recently, investigators from the Genetic Risk and Outcome of Psychosis (GROUP) collaboration studied a large sample of patients with a psychotic disorder (*n* = 1120), their siblings (*n* = 1057), and community controls (*n* = 590). In this prospective, ongoing study, the investigators found that the effect size of the relationship between current cannabis use and both positive and negative schizotypy symptoms was significantly greater in siblings of patients with a psychotic disorder when compared to healthy, un-related control. Further, there was a significant association between cannabis-using siblings and their psychotic patient relatives (in terms of positive symptoms), whereas this association did not emerge among non-exposed siblings and their psychotic relatives. The authors proposed that the familial liability to psychosis is expressed partially in terms of psychotomimetic experiences with cannabis (GROUP).

### History of childhood abuse

More recently, the interactive effects of childhood maltreatment and cannabis abuse have been examined. In a cross-sectional study, Houston and colleagues found an odds ratio of 11.96 (95% CI 2.10–68.22) for having experienced psychosis among children with a history of abuse who used cannabis prior to age 16 (275). Another cross-sectional study by Harley et al. found a significant interactive effect of childhood trauma and cannabis use in moderating the risk of psychotic symptoms; the odds ratio of experiencing psychosis in adolescents with a history of exposure to trauma and cannabis was 20.9 (95% CI 2.3–173.5) (276). A longitudinal study has similarly shown a significant interaction between cannabis use and childhood maltreatment in the development of psychotic symptoms (277). Notably, there was no evidence in this study that baseline history of childhood abuse affected subsequent cannabis use. These findings, however, were not replicated in the EDSP dataset (278). It is important to interpret the above findings with caution. Some investigators (279) have shown a link between childhood abuse and subsequent cannabis use; others demonstrate a link between abuse and subsequent psychosis (280). Future studies, which examine the interaction between genetic liability, trait measures of psychosis liability, cannabis use, and other environmental factors may provide greater insights into the complex mechanisms that cause psychosis.

### Genetic factors

Genetic factors may confer vulnerability to psychosis outcomes following exposure to cannabis, i.e., a gene-environment interaction. In specific, Catechol-*O*-methyltransferase (COMT) and *AKT1*, have been implicated in conferring such vulnerability (see Table 1). Preliminary evidence suggests that other genes might also moderate the cannabis–psychosis interaction.

**Table 1:** **Gene × cannabis interactions in moderating risk of psychosis**.

Gene/locus	Study	Study design	Sample size	Follow up	Outcome – odds ratio (OR)/relative risk (RR)
*COMT*/rs4680	Caspi et al. (200)	Longitudinal, prospective (Dunedin cohort)	803	26 years	OR 10.9 (95% CI 2.2–54.1) of developing psychotic disorder in Val/Val genotype
					OR 2.5 (95% CI 0.78–8.2) of developing psychotic disorder in Val/Met allele
					OR 1.1 (95% CI 0.21–5.4) of developing psychotic disorder in Met/Met allele
*COMT*/rs4680	Zammit et al. (281)	Case-only, cross-sectional analysis	493	NA	OR 0.98 (95% CI 0.76–1.27) for history of cannabis use in schizophrenia subjects with Val/Val allele
*COMT*/rs4680	Zammit et al. (282)	Longitudinal (Avon cohort)	2630	2 years	OR 1.0 (95% CI 0.73–1.36) of cannabis × COMT interaction
					OR 1.56 (95% CI 1.05–2.31) of psychosis in cannabis users with Met/Met genotype
					OR 1.47(95% CI 0.85–2.26) of psychosis in cannabis users with Val/Val genotype
					OR 1.68 (95% CI 1.23–2.28) of psychosis in cannabis users with Met/Val genotype
*COMT*/rs4680	Costas et al. (283)	Case-only, cross-sectional analysis	748	NA	OR 2.07 (95% CI 1.27–3.26) of history of cannabis use in schizophrenia pts w/Met/Met genotype vs. Val/Val genotype
*AKT1*/rs2494732	van Winkel (284)	Cross-sectional analysis	801 Subjects with psychosis	NA	RR 1.90 (*p* < 0.01) of C/C genotype in daily cannabis users – case-only analysis
			740 Unaffected siblings		OR 1.96 (95% CI 1.09–3.53) of being diagnosed with psychotic disorder in C/C allele subjects – case–sibling analysis
			419 Controls		OR 2.08 (95% CI 0.92–4.67) of being diagnosed with psychotic disorder in C/C allele subjects – case–control analysis
*AKT1*/rs2494732	Di Forti et al. (285)	Case–control, cross-sectional analysis	489 Subjects	NA	OR 7.23 (95% CI 1.37–38.12) of psychotic disorder in C/C genotype subjects with daily cannabis use vs. T/T genotype
			278 Controls		OR 2.18 (95% CI 1.12–4.31) of psychotic disorder in C/C genotype subjects with history of cannabis use

*OR, odds ratio; RR, relative risk; CI, confidence interval*.

#### Catechol-*O*-methyltransferase

In one of the first studies that drew attention to gene × environment interactions, Caspi et al. reported that the *COMT* gene moderated the risk of psychotic disorder with adolescent cannabis exposure. The enzyme COMT plays a critical role in the breakdown of dopamine in the PFC (286), in contrast to the striatum where DA is cleared by a transporter. The *COMT* gene has a common polymorphism in humans, which results in 40% higher enzymatic activity and thus more rapid degradation of dopamine when Valine (107) is substituted for Methionine (Met) at the 158/108 locus. Val/Val homozygotes have the lowest levels of dopamine; Met/Met homozygotes have the highest levels; and heterozygotes have intermediate levels. Lower cortical dopamine levels in individuals homozygous for the Val(158) polymorphism are associated with, among other things, poorer cognitive performance, and inefficient precortical functioning (287).

In a longitudinal prospective study (Dunedin cohort) of 803 individuals followed over 25 years, Caspi et al. showed that the risk of developing of psychotic disorder in association with cannabis exposure increased by 10-fold in those patients with the Val/Val allele (200). There were subsequent attempts to validate these findings with experimental evidence: a double-blind, placebo-controlled cross-over study showed that individuals with the Val polymorphism of the *COMT* gene have a higher chance of developing acute psychosis in response to THC exposure (133). These findings have been confirmed in a similar experiment (288).

Recent studies have failed to confirm the findings of the original 2005 study from Caspi and colleagues. A case-only analysis of 1438 individuals found no interaction between *COMT* polymorphism and cannabis use with regard to schizophrenia (281). Further, a 2-year longitudinal study of 2630 genotyped patients showed no interaction between *COMT* and cumulative cannabis use on the development of psychosis (282). A more recent case–control study also showed no *COMT-*mediated increased cannabis risk in the development of psychosis (284). Kantrowitz et al. were unable to find an interaction between COMT polymorphisms and cannabis-induced psychotic disorder in a population of 92 individuals with psychotic disorder, though this study was under-powered. Sub-analyses based on race (African American and Caucasian) did not yield significant findings (289). In contrast to the original Caspi et al. study (200), a case-only study from Spain (155 out of 748 total schizophrenia subjects who used cannabis) actually found an association between the low-activity Met allele of *COMT* and cannabis use in psychotic disorder (283). Estrada et al. (290) showed a dose-effect of *COMT* polymorphism on the age of onset of psychosis among cannabis users: individuals who were homozygous for the Val allele of COMT had the earliest age of onset of psychotic disorders at 15.4 years; homozygotes for the Met allele had the latest age of onset at 18.8 years; heterozygotes with intermediate enzymatic activity, had an age of onset of 17.1 years. Notwithstanding, there was no overall greater risk for psychotic disorder found among any of the polymorphism groups. A similar trend regarding the interaction of *COMT* polymorphism and cannabis use in association with the age of onset of psychosis has been shown, though not all results achieved statistical significance (291).

Other studies have examined the interactive effects of *COMT* polymorphisms and other environmental factors. A cross-sectional analysis of 918 individuals in Europe found a significant three-way interaction between the *COMT* Val allele, cannabis use, and childhood abuse in moderating psychosis. Individuals homozygous for the Val polymorphism were more likely to experience psychosis in association with cannabis use in the context of a history of childhood abuse than individuals homozygous or heterozygous for the Met allele. A replicative sample as part of the same study showed the same trend but did not achieve statistical significance (292). Confirming these findings, Alemany et al. found that the three-way interaction of *COMT* polymorphism (Val allele), cannabis use, and a positive history of child abuse significantly increased the risk of both positive and negative psychotic symptoms (293).

#### AKT1

*AKT1* is another gene thought to play a role in moderating the association between cannabis and psychotic disorders. The enzyme AKT1 functions to inactivate glycogen synthase kinase (GSK-3) by phosphorylation (294). The interaction between AKT1 and GSK-3 has been implicated to play a role in a number of important cellular processes, such as cell proliferation, apoptosis, and transcription (295). *In vitro* studies have shown that cannabinoids are capable of stimulating the AKT1 pathway via CB_1_ and CB_2_ receptors (296) and *in vivo* studies in mice have also confirmed this (297). Further, the gene product has been implicated in schizophrenia: postmortem studies have shown decreased AKT1 levels in lymphoblasts in the PFC of patients with schizophrenia (298, 299).

In a sample comprised of 801 patients with psychosis, 740 of their unaffected siblings, and 419 controls, van Winkel showed that cannabis users with the C/C genotype of a specific polymorphism (rs2494732) of the *AKT1* gene had a twofold increase in risk of being diagnosed with psychotic disorder (284). Additionally, among psychotic patients, those homozygous for the C allele were twice as likely to have a history of daily cannabis use compared with T/T genotypes. The significance of the AKT1 × cannabis interaction held among case-only (*p* = 0.007) and case–sibling (*p* = 0.04) sub-analyses; in the case–control sub-analysis, the AKT1 × cannabis interaction approached statistical significance (*p* = 0.057). A more recent study has replicated these findings and found an even stronger interaction. Di Forti and colleagues studied 489 patients with FEP and 278 control subjects in a case–control design; among daily cannabis users, those who carried the C/C allele had, on average, a sevenfold increase in the risk of psychosis compared to T/T carriers (285). Notably, carriers of this genotype (C/C at SNP rs2494732) also have been shown to have increased cognitive side effects from cannabis use as evidenced by lower scores on tests of sustained attention (300). Preliminary experimental evidence has also implicated a different polymorphism of the *AKT1* gene (the GG genotype of the SNP rs1130233) as a moderator of sensitivity to the acute psychosis-inducing effect of THC (301).

#### Other genes

Another gene implicated in moderating the effects of cannabis on the development of psychosis is *DAT1*, which codes for the dopamine transporter, which is critical in removing DA from the synapse in striatal regions. A polymorphism involving a variable number of tandem repeats (VNTR) has been described in the 3′ untranslated region of the *DAT1* gene (SLC6A3). One of the common alleles of this polymorphism (the nine-repeat allele) is associated with lower enzymatic activity and thus higher dopamine levels in the striatum. *DAT1* has previously been associated with schizophrenia (independent of cannabis use) in gene association studies (302). Bhattacharyya et al., reported that individuals with the nine-repeat allele showed increased sensitivity to THC-induced psychotomimetic effects in a small laboratory based study (*n* = 35) (301). There was also a trend toward greater THC-induced psychotomimetic effects in individuals with the G/G genotype of the rs1130233 polymorphism of the *AKT1* gene in the same sample. Furthermore, there was a synergistic interaction between these *DAT* and *AKT1* genotypes on the psychotomimetic effects of THC. In addition to studying behavioral effects of THC, this study showed interactive effects of *DAT1* genotype, *AKT1* genotype, and THC on striatal and midbrain activation during encoding and recall of verbal information, respectively. Individuals with the GG allele at *AKT1* and carriers of the nine-repeat allele of *DAT1* also showed increased activation in the striatum in response to THC in comparison to the rest.

Neuregulin 1 (*NRG1*), a leading schizophrenia susceptibility gene, is relevant to several schizophrenia-related neurodevelopmental processes (303, 304). Heterozygous deletion of *NRG1* results in increased sensitivity of mice to schizophrenia-like symptoms induced by THC especially under stressful conditions (305). These mice also showed greater increases in prepulse inhibition (PPI), a marker for sensorimotor gating known to be impaired in schizophrenia, following THC administration (305). However, to our knowledge, this work has not yet been extended to humans. Decoster et al. reported significant interactions between brain-derived neurotrophic factor (*BDNF*) genotype, cannabis exposure, and gender in a cohort of schizophrenia patients: in female patients only, cannabis use was associated with earlier age of onset of psychosis in *BDNF* Met-carriers relative to Val/Val-genotypes (306). Additionally, cannabis use may interact with specific genotypes of the cannabinoid receptor 1 (*CNR1*) gene to moderate cognitive impairment in schizophrenia patients (307), but thus far no significant interaction between *CNR1* polymorphisms and cannabis exposure on the risk for the development of psychotic disorders has been reported (281).

## Cannabis, Schizophrenia, and Causality

The association between cannabis and psychosis fulfills many but not all of the standard criteria for causality (308), namely temporal relationship, biological gradient, biological plausibility, coherence, consistency, and experimental evidence.

### Temporal relationship

As discussed above, evidence from experimental studies shows a clear temporal relationship between exposure to cannabinoids and symptoms of psychosis. Despite a number of limitations (discussed previously), several epidemiological studies have concluded that cannabis use generally precedes the development of psychotic disorder. In one of the earliest such studies, Allebeck and colleagues found that cannabis use preceded the onset of schizophrenia by at least 1 year in 69% of cases; in only 11% of cases did cannabis succeed psychosis (309). In a prospective cohort study, Linszen et al. found that in all but 1 patient from a sample of 24 cannabis-abusing patients, cannabis abuse preceded FEP by at least 1 year (310).

Studies from recent years suggest that in the majority of cases, cannabis use precedes the onset of psychosis, rather than vice versa. In a study of 28 FEP patients, cannabis use preceded psychosis in all patients (267). Another study of 45 psychotic disorder patients with a history of cannabis use showed that the onset of cannabis use preceded hallucinations in 74% of cases and preceded persecutory ideas in 90% of cases by at least on year (250). Schimmelmann and associates (251) reported that in 88% of cases (*n* = 201 FEP patients with cannabis use), drug exposure preceded psychotic symptoms by a mean of 5 years.

As discussed above, numerous additional studies have shown that cannabis users have a younger age of onset of psychotic disorders compared to non-users (197, 250, 258, 309–312). A recent meta-analysis of over 22,000 subjects found the onset of psychosis was 2.7 years younger in cannabis users than in non-users (252). These studies lend further evidence to the finding that cannabis usually precedes the onset of psychotic symptoms and argue against the “self-medication” hypothesis.

The findings from epidemiological studies regarding the temporal relationship between cannabis and psychosis must be qualified. Epidemiological studies have traditionally examined the relationship of cannabis use and psychosis as defined by positive psychotic symptoms. It is unclear whether the same temporal relationship holds for cognitive deficits or negative symptoms of psychosis, which usually predate the onset of positive psychotic symptoms. Furthermore, the data fails to explain why patients with schizophrenia continue to abuse cannabis. Cannabis continues to be among the most common illicit drug used by patients with schizophrenia. In the Australian Study of High Impact Psychoses (313), 49% of patients with schizophrenia reported exposure to cannabis in the past year (314). In a study among patients with schizophrenia using experience-sampling, Henquet et al. (315) found that compared to healthy control, patient with schizophrenia reported a reduction in negative affect after cannabis use, while the increase in positive affect that they experienced was comparable to controls. Schizophrenia is a disease of gradual onset and diagnosis usually occurs only when a patient’s symptoms are severe enough to cause disruptions in psychosocial functioning. If, as has been hypothesized, schizophrenia is a neurodevelopmental disorder in which neurobiological changes occur years before the onset of symptoms, then these studies have been unable to examine the true temporal relationship between psychosis and cannabis. On the other hand, if cannabis induces schizophrenia in individuals who are genetically vulnerable (see discussion below) and thus exhibit “prodromal” symptoms at baseline, then the exact temporal nature of this relationship is extremely relevant.

### Biological gradient

There are a number of limitations to assessing the dose of exposure of cannabis and its effect on psychotic outcome. Whereas cigarettes and alcoholic beverages have standardized and well-known quantities of nicotine and alcohol, the THC content of cannabis varies considerably. Further, when people smoke cannabis, they may smoke the same joint over several sessions or share a joint with others. Therefore, the number of times a person has smoked is a crude proxy of the “dose” of cannabis exposure. Finally, as discussed above, CBD is thought to have antipsychotic effects in opposition to the pro-psychotic effects of THC. The variable content of CBD in marijuana further complicates the interpretation of studies investigating a dose–response effect.

Despite these limitations, a consistent dose–response effect has been shown in numerous studies. One of the earliest studies showing a biological gradient in the association between cannabis use and psychotic symptoms was done by Andreasson and colleagues. Using the Swedish military conscript (*n* > 45,000) followed over a 15-year period (described in detail previously), the investigators found that individuals with heavier cannabis use (>50 occasions of consumption) had a greater chance of developing schizophrenia (relative risk 6.0); intermediate users (11–50 occasions of consumption) had a relative risk of 3.0 for developing schizophrenia. After adjusting for various potential confounders (school adjustment, socio-economic status, solvent abuse, psychiatric diagnosis or medications at baseline, and others, but not including childhood abuse/trauma), the relative risk remained elevated and statistically significant (197). A follow up of this same cohort at 27 years found that this dose–response relationship between cannabis consumption and risk of developing schizophrenia persisted over time (198). Other studies previously described, including the NEMESIS (20) and the ESDP cohorts (24) have also suggested a biological gradient between exposure load and psychotic outcome. More recent evidence also supports this dose–response effect in a sample of individuals with sub-clinical psychotic symptoms; this sample showed that among heaviest users (>5 per day) the relative risk, after adjusting for confounders (“sex, age, social exclusion, alcohol, cannabis use before age 17, and heavy non-cannabis drug use”), of experiencing auditory hallucinations was 5.4 and relative risk for first-rank symptoms was 11.6 (316).

### Specificity

The specificity of the association between cannabis and psychotic disorders is low. In a prospective study of 3-year follow up, of all patients who developed psychosis (assessed by BPRS), only 21% had any use of cannabis at baseline. Furthermore, of those who used cannabis at baseline, only 8 in 312 subjects (2.6%) developed psychosis (20). Similar data was reported from the Swedish military conscript cohort (197). While the association between cannabis and schizophrenia is not specific, it is stronger and more consistent than the association between cannabis and anxiety or depressive disorders. Odds ratios for the development of anxiety or depressive disorders with exposure to cannabis typically range from 0.7 to 1.5 with many studies yielding statistically insignificant results; in contrast, a meta-analysis of multiple longitudinal prospective studies found a statistically significant, adjusted odds ratio of 2.09 (95% CI 1.54–2.84) for psychosis outcome among heaviest cannabis users with all but one of the six high-quality, longitudinal studies showing a statistically significant outcome. These longitudinal studies controlled for about 60 different potential confounders, including personality traits, socio-economic markers, other substance use, and other mental health problems (25). In a longitudinal study of over 18,000 patients hospitalized for substance-induced psychosis, the 8-year cumulative risk of conversion to schizophrenia was 46% when the offending substance was cannabis. In contrast, the conversion rate to schizophrenia over the same period of time for alcohol-induced psychosis was 5%. Notably, the risk for the development of schizophrenia when the diagnosis was amphetamine-induced psychosis was 30% (188).

### Consistency

While not all epidemiological studies have detected an association between cannabis use and psychosis, most longitudinal studies (described in detail previously) show a statistically significant increased risk of psychosis outcome in those who use cannabis heavily. Among each study’s heaviest users, the following longitudinal studies have demonstrated a significantly increased risk of any psychosis outcome: the Swedish military conscript cohort (heaviest users being those who had used marijuana >50 times) (197–199), the NEMESIS cohort (weekly users) (20), EDSP cohort (daily users) (24), Epidemiological Catchment Area study (daily users) (317), Dunedin cohort (19, 200), and the CDHS cohort (daily users) (23). The National Psychiatric Morbidity Survey found an increased odds ratio (adjusted for alcohol consumption, gender, IQ score, marital status, and others) that was not statistically significant (even in their heaviest using subjects, those with cannabis dependence) (318). Among these same studies, an analysis of those who had ever used marijuana (even if just once), the Epidemiological Catchment Area study, EDSP study, and the NEMESIS cohort showed increased risk of any psychosis outcome but this risk was not statistically significant.

### Biological plausibility

The precise pathophysiology of psychosis or psychotic disorders remains unclear; therefore, a biologically plausible mechanism whereby exposure to cannabis can increase the risk for psychosis or a psychotic disorder is yet to be established. THC, the principal active component of cannabis, is a partial agonist at CB_1_Rs where it has modest affinity (*K*_i_ = 35–80 nmol) and low intrinsic activity (319). CB_1_Rs are G-protein-mediated receptors that are distributed with high density in the cerebral cortex (particularly frontal regions), basal ganglia, hippocampus, anterior cingulate cortex, and cerebellum; these brain regions have been implicated in the putative neural circuitry of psychosis. The primary effect of cannabinoids is the modulation of neurotransmitter release via activation of presynaptic CB_1_Rs. Thus cannabinoids, by activating CB_1_Rs, can modulate the release of a number of neurotransmitters already implicated in psychosis, including dopamine, glutamate or GABA.

The dopamine hypothesis, which postulates that positive symptoms of psychosis may be attributed to disturbed and hyperactive dopaminergic activity, remains one of the more enduring and dominant hypotheses of schizophrenia (320). CB_1_R-mediated increases in mesolimbic dopaminergic activity may explain the positive psychotic symptoms induced by THC. Converging preclinical evidence suggests interactions between cannabinoid (CB_1_) and dopamine (DA) systems [reviewed in Ref. (321, 322)]. CB_1_ and D_2_ receptors are co-expressed in several brain regions (323) and there is signal transduction convergence in these regions (324). Cannabinoids have been shown to induce firing of dopaminergic mesolimbic neurons and induce DA release in the striatum in animals (100, 321, 325–329). Cannabinoids regulate DA firing via a CB_1_-GABAergic-mediated disinhibition of DA neuronal activation. However, the results of *in vivo* imaging studies of THC-induced striatal dopamine release in humans have been mixed (96, 330–332). The effect of cannabinoids on striatal dopamine release may be differentially affected by biological vulnerability for psychosis. While chronic cannabis use was found to be associated with decreased striatal dopamine synthesis in healthy individuals (332), THC was found to increase striatal dopamine release in first-degree relatives of individuals with psychotic disorder (333).

The effects of cannabinoids on dopaminergic systems in the PFC might account for some of their acute cognitive deficits. It is well-know that either too much or too little dopaminergic activity in the PFC is associated with impairments in PFC-related cognitive functions leading to an inverted “U” (bell shaped) relationship between dopamine levels and working memory efficiency (334, 335). Systemic administration of cannabinoids has been reported to increase prefrontal cortical DA release or turnover in several studies (100, 336–339). This may explain how cannabinoids produce acute impairments in PFC-related cognitive functions including working memory and attention.

Cannabinoids might induce psychosis and cognitive impairments via actions on GABAergic systems. Higher order cognitive processes, including working memory, are associated with θ (4–7 Hz) and γ (30–80 Hz) oscillations in the PFC. Deficits in working memory are a hallmark of schizophrenia and are associated with reduced cortical θ and γ band power. Cortical θ and γ oscillations are dependent on inhibition of pyramidal neurons. This inhibition is driven by specific cholecystokinin (CCKb cells) and parvalbumin (PVb cells) containing GABAergic interneurons. In several brain regions, CB_1_Rs are present on the terminals of axons in cholecystokinin (CCK)-containing GABA interneurons that target the perisomatic regions of pyramidal cells (340). Activation of CB_1_R reduces GABA release, which in turn releases the inhibition effects on pyramidal cells. While admittedly speculative, the disinhibition of pyramidal cells may lead to cortical oscillation deficits and working memory impairments.

While the acute effects of cannabinoids on DA, GABA, and glutamate neurotransmission may explain some of the acute positive, negative, and cognitive symptoms of cannabinoids, the mechanism by which exposure to cannabinoids might cause schizophrenia has not yet been established. If schizophrenia is a neurodevelopmental illness (341, 342), then the observation that early cannabis exposure is associated with a greater risk for the development of schizophrenia may offer some clues to the underlying biological mechanisms. Consistent with the human epidemiological data, animal studies suggest that early (adolescent) but not later (adult) exposure to cannabinoids is associated with persistent impaired social behaviors, including psychotic-like behaviors, cognitive, and sensorimotor gating deficits in adults (253–257).

Adolescence and young adulthood are critical phases for cerebral development. Brain development continues into young adulthood (up to 25 years) (343) and therefore, any factors that interfere with brain development during this time may have far reaching consequences. During this period of neuronal plasticity, there is sprouting and pruning of synapses, myelinization, changes in neurotransmitter concentrations and their receptor levels in brain areas necessary for behavioral and cognitive functions (344). The endocannabinoid system plays an important role in several processes important in neurodevelopment including neurogenesis, neural specification, neural maturation, neuronal migration, axonal elongation, glia formation, and positioning of inhibitory GABAergic interneurons and excitatory glutamatergic neurons (259–262, 345–349). Perturbation of the endocannabinoid system in the rapidly changing brain, as is the case in adolescence, by excessive or non-physiological stimulation, as may be the case with exposure to exogenous cannabinoids, may have far reaching consequences. This would be especially so in the presence of already altered neurodevelopmental processes. Therefore, by disrupting the endocannabinoid system and interfering with neurodevelopmental processes, exogenous cannabinoids may provide a biologically plausible mechanism by which exposure to cannabinoids during adolescence may increase the risk for the development of schizophrenia.

### Strength of association

In the general population, the strength of association between any cannabis exposure and the development of psychosis is modest. A systematic review of 35 longitudinal studies found the relative risk of developing schizophrenia after any cannabis exposure to be 1.4 after adjusting for about 60 potentially confounding variables, including personality traits, socio-economic markers, other substance use, and other mental health problems (25). However, as discussed above, in heavy users (as well as those who begin use at earlier ages), the risk can be much greater. A follow up of the original Swedish military conscript after 35 years yielded an adjusted relative risk of 3.7 for the development of a psychotic disorder (199).

Indirect but compelling evidence is seen in conversion of cannabis-induced psychosis to schizophrenia. Longitudinal studies have found that the risk of developing schizophrenia is nearly 50% in patients admitted for cannabis-induced psychosis (181, 188). Such findings suggest that genetic (or other predisposing) susceptibility to cannabis-induced psychosis may explain why the cannabis–schizophrenia association does not fulfill all causality criteria. That is, in a sub-population of individuals with a history of childhood abuse and genetic vulnerability, the association between cannabis and schizophrenia may be significantly stronger and more specific than in the general population. Individuals with neurobiological vulnerabilities who develop acute psychosis, which persists for a limited period after cannabis intoxication may be those who, with prolonged exposure, are more likely to develop permanent psychotic disorders.

### Experimental evidence

As noted above, direct experimental evidence for acute and transient psychosis caused by cannabis intoxication is compelling (89, 95). In some individuals, this effect persists after the acute intoxication period has ended. In randomized, placebo-controlled, experimental settings, acute psychosis in response to THC intoxication is quite common and reproducible (89, 95, 350). Positive (paranoia, grandiose delusions, fragmented thinking, and perceptual alterations), negative (blunted affect, emotional withdrawal, and psychomotor slowing), and cognitive symptoms (impairments of abstraction, attention, executive function, and memory) have been well-documented. Thus, the main symptom clusters of schizophrenia are seen acutely with THC intoxication. Occasionally, immediate psychosis precipitated by cannabis persists beyond the period of intoxication and may require intervention, though most of these data come from case reports and small series rather than experimental evidence.

Unlike studying acute effects, an experimental approach to characterize the effects of chronic, heavy, and early cannabis exposure is neither ethical nor feasible. An alternative approach is to compare a group with chronic, heavy early cannabis use to controls. Such samples do exist and have been discussed in detail previously.

### Coherence

There is substantial coherence between the laboratory study findings and epidemiological findings regarding the acute effects of cannabinoids. Cannabinoids induce a range of psychosis-like effects in laboratory studies and epidemiological studies are replete with reports of psychosis following the consumption of cannabinoids. Similarly, cannabinoids have been shown to exacerbate symptoms in individuals with a psychotic disorder and epidemiological studies have shown that cannabis use by schizophrenia patients is associated with a negative impact on the expression and course of the illness. However, as an experimental approach to characterize the effects of chronic, heavy, and early cannabis exposure is neither ethical nor feasible, it is impossible to determine coherence between laboratory and epidemiological studies with regard to the consequences of chronic, early, and heavy cannabis use and psychosis.

### Parallels

Several parallels can be drawn between the cannabis–psychosis association and other associations in medicine that have been accepted to be causal in nature. For instance, excess salt consumption has been shown to be a well-established cause of hypertension (351), yet not all people who consume more than 2 g of salt daily have hypertension. Similarly, most people who smoke cigarettes do not develop lung cancer; further, there are types of lung cancer (i.e., adenocarcinoma), which develop in the absence of smoking. Yet smoking is understood to be the single most important modifiable causal component in the development of lung cancer.

It is unlikely that schizophrenia is a homogenous disorder with a single pathophysiology; instead, it is more likely a syndrome with distinct neurobiological etiologies. Similarly, the term “lung cancer” comprises several different histological types, including adenocarcinoma, squamous cell carcinoma, and small-cell carcinoma. The risk that smoking confers in the development of cancer varies considerably as the sub-type of cancer becomes more specific. For instance, the risk of developing various types of cancer (including liver, kidney, cervical, myeloid leukemia, gastric, nasopharyngeal, nasal, or esophageal adenocarcinoma) among current smokers may be relatively low, with estimates of the relative risk being ~1.5–2.0 (352). In a large meta-analysis, the relative risk of developing any *lung* cancer among current smokers is much higher at 8.43 (95% CI 7.63–9.31); the relative risk of developing squamous cell carcinoma of the lung is even higher, recently cited at 16.43 (95% CI 12.66–21.32) (353). Viewed from another perspective of the analogy, it is estimated that tobacco smoke is responsible for ~21% of all types of cancer-related deaths worldwide (354) and 87% of all deaths related to lung cancer (2013). By comparison, it is estimated that 8–14% cases of schizophrenia may be due to cannabis use (25, 207). Therefore, the magnitude of the risk for schizophrenia conferred by cannabis exposure is significantly lower than the risk of lung cancer conferred by smoking. It is unlikely that there is any single cause of an illness as heterogeneous as schizophrenia. As research progresses and our understanding of the biological causes of mental illness advances, cannabis-induced psychotic disorder may emerge as a distinct sub-type among the different disorders that constitute what we now classify broadly as schizophrenia.

In summary, the relationship between cannabinoids and psychosis fulfills many but not all of the traditional criteria for causality. Given the evidence presented above, it is likely that cannabis is an important component cause in the development of psychotic disorders (16, 205). This causal role is likely magnified when cannabis exposure occurs at an earlier age, in greater quantities, and over a longer time-course. Further, as discussed elsewhere in this review, specific populations (i.e., those with a genetic vulnerability or a history of childhood abuse) may be particularly susceptible to the causal effects of cannabis. Notably, although meta-analytical studies suggest that cannabis might account for between 8 and 14% of schizophrenia cases (25, 207), the fourfold increase in the rates of cannabis use over the last four decades (198, 355) has not resulted in a commensurate 40–70% increase in prevalence of schizophrenia. Some studies suggest that the rates of schizophrenia may be decreasing (356), while others suggest the contrary (357, 358). The discrepancy between the recent changes in the rates of cannabis consumption and relative stability of schizophrenia rates are difficult to explain in the context of the findings reviewed above; one possible explanation is that schizophrenia rates are lagging behind increased rates of cannabis consumption. Again, it is important to note that schizophrenia is likely a very heterogeneous illness, comprised of multiple sub-types. It is unlikely that there is a single causative factor. As proposed by Rounsaville, it is possible that a cannabis-induced psychotic disorder comprises one of the distinct sub-types of the schizophrenia-spectrum (191).

## Summary and Conclusion

In summary, acute exposure to both natural and synthetic cannabinoids can produce a full range of transient symptoms, cognitive deficits, and psychophysiological abnormalities that bear a striking resemblance to some of the features of schizophrenia. Also clear is that, in individuals with an established psychotic disorder, cannabinoids can exacerbate symptoms, trigger relapse, and have negative consequences on the course of the illness. Finally, exposure to cannabinoids in adolescence confers a higher risk for psychosis outcomes in later life and the risk is dose-related. However, it should be remembered that the majority of individuals who consume cannabis do not experience any kind of psychosis.

The findings from research reviewed above have profound implications for public health. Aside from alcohol, cannabis is currently the most prevalent drug used worldwide. In the United States, the legal status of cannabis for medical and recreational purposes is changing rapidly. Pertinent findings that are likely to impact public health include high conversion rates from cannabis-induced psychosis to schizophrenia; global and specific domains of cognitive impairment resulting from cannabis use, which may be irreversible; the effects of acute intoxication; the precipitation of psychotic disorders in genetically vulnerable populations, including individuals with a history of childhood abuse or family history of psychotic disorders; and the increased risk of negative effects of cannabis use in prolonged and early exposure. Additional high-quality epidemiological studies are needed to further characterize the extent to which cannabis causes these negative effects or unmasks them in a vulnerable subset of the population.

## Conflict of Interest Statement

The authors declare that the research was conducted in the absence of any commercial or financial relationships that could be construed as a potential conflict of interest.
